# Trends in selected fields of reference material production

**DOI:** 10.1007/s00216-022-03996-7

**Published:** 2022-03-22

**Authors:** Sebastian Recknagel, Harald Bresch, Heinrich Kipphardt, Matthias Koch, Martin Rosner, Ute Resch-Genger

**Affiliations:** 1 Bundesanstalt für Materialforschung und -prüfung (BAM), Richard-Willstätter-Straße 11, 12489 Berlin, Germany; 2 IsoAnalysis UG, Volmerstr. 7a, 12489 Berlin, Germany

**Keywords:** Reference material, ISO REMCO, Gas analysis, Food, Nanomaterials, Fluorescence

## Abstract

For more than 110 years, BAM has been producing reference materials for a wide range of application fields. With the development of new analytical methods and new applications as well as continuously emerging more stringent requirements of laboratory accreditation with regard to quality control and metrological traceability, the demand and requirements for reference materials are increasing. This trend article gives an overview of general developments in the field of reference materials as well as developments in selected fields of application in which BAM is active. This includes inorganic and metal analysis, gas analysis, food and consumer products, and geological samples. In addition to these more traditional fields of application, developments in the areas of optical spectroscopy, particularly fluorescence methods, and nanomaterials are considered.

## Introduction

At the turn of the nineteenth and twentieth centuries, when industrial production grew very strongly, there emerged a growing need for tools to back up chemical-analytical test results. This prompted the National Bureau of Standards (NBS) in the USA to launch the first cast iron reference materials in 1906. Only 6 years later—in 1912—the Royal Materials Testing Office in Berlin-Dahlem, the predecessor organization of BAM, issued the first standard steel for carbon determination. In 1916, also in the UK, reference material production started with the British Chemical Standards (BCS) program followed by the Societé Française des Echantillons-Type in France in 1922 [1].

Meanwhile, an increasing number of reference materials (RMs) and certified reference materials (CRMs) has become available for measurements relevant to many fields of application. For example, CRMs for chemical composition are used (1) to assess the accuracy or trueness of measurement results, (2) to assist in the validation of analytical methods, (3) to serve as control materials for quality assurance of routine analyses, (4) to provide metrological traceability of measurement results, (5) to serve as calibrants, and (6) to serve to estimate measurement uncertainty.

Technological progress is a general driver for CRM production. The development of new analytical methods and new analytical challenges trigger the demand for RMs. In addition, also the development and availability of reference methods and reference data, e.g., validation data sets, are becoming increasingly important. Another important driver for the production and use of RMs and CRMs is the fact that testing and calibration laboratories accredited according to ISO/IEC 17025 are obliged to use CRMs for quality control (QC) and to validate measurements through traceable results with stated uncertainties [2, 3]. Also, accreditation not only of testing laboratories but also of RM producers according to ISO 17034 [4] becomes more and more common. Meanwhile, in Germany, there are 21 accredited RM producers, six of them since 2021 [5]. Worldwide, about 200 RM producers have an International Laboratory Accreditation Cooperation (ILAC) recognized accreditation [6].

**Fig. 1:**
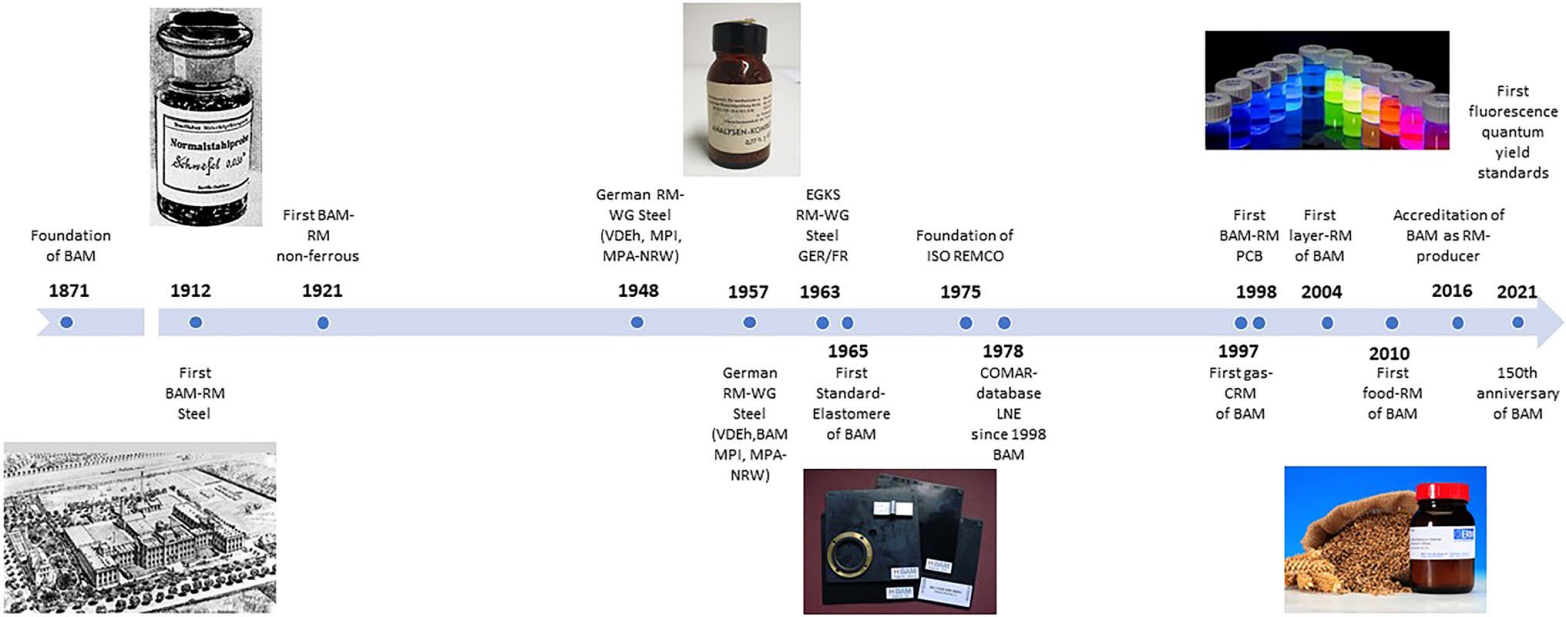
Historical overview of the RM production at BAM (abbreviations: VDEh—Verein deutscher Eisenhüttenleute; MPI—Max-Planck-Institut für Eisenforschung; MPA-NRW—Materialprüfungsamt Nordrhein-Westfalen; EGKS—Europäische Gemeinschaft für Kohle und Stahl; ISO—International Standardisation Organisation; REMCO—reference materials comittee; COMAR—Code d'Indexation des Matériaux de Référence; PCB polychlorinated biphenyls)

In the following, we will give an overview on trends in RM production in general as well as for selected application fields representative of RM activities of the Bundesanstalt für Materialforschung und -prüfung (BAM). A historical overview of the CRM and RM production at BAM and selected CRMs for different areas of application is given in Fig. 1.

## Development of RM preparation

The classical RM hierarchy described in ISO Guide 30 [7] distinguishes between RM, a “*material, sufficiently homogeneous and stable with respect to one or more specified properties, which has been established to be fit for its intended use in a measurement process*,” and CRM, a “*reference material (RM) characterized by a metrologically valid procedure for one or more specified properties, accompanied by an RM certificate that provides the value of the specified property, its associated uncertainty, and a statement of metrological traceability*.” In addition, definitions are given for primary and secondary measurement standards. From a metrological point of view, primary standards are at the top of the metrology pyramid and embody the origin of the metrological traceability chain. For inorganic analysis, these can be, for example, ultrapure metals certified for the content of the main constituent [8]. CRMs are defined as—related to the parameter of interest—homogeneous and stable materials with certified properties (e.g., elemental content) traceable to the Système international d’unités (SI) by means of primary standards, for which an uncertainty is stated. These CRMs are produced with great effort. RMs, on the other hand, are characterized as homogeneous and stable materials traceable to CRMs. In addition to these classic RMs (used here as a generic term or “family name” [9]), where CRMs form a sub-group, more simple types of RMs are emerging that are more rapidly available and less expensive than CRMs. Examples for these “fit-for-purpose materials” are NIST-defined Research-Grade Test Materials (RGTMs). RGTMs form a subset of the “exploratory materials” that NIST provides for new measurement tasks [10]. They are prepared to be homogeneous and stable for a particular purpose but are typically not fully characterized with respect to all properties of potential interest. Triggered by a sufficient interest of the scientific and industrial community and utilizing published data, these RGTMs may later become CRMs. This interaction between producer and user of a CRM can have a considerable influence on an existing CRM as well. This is already common practice in the case of geological RMs [11].

CRM producers normally use state-of-the-art analytical techniques and approaches to assign the best estimate of the true value. A certified value for a CRM can change over time as the analytical methods including sample pretreatment steps and/or certification approaches improve, i.e., in sensitivity. This does not mean that the original value was “wrong” at the time of assignment. Instead, the analytical methods and the understanding of the measurement system have evolved over time, enabling, e.g., a (re)certification with a lower uncertainty. If different results are found using an advanced analytical method or approach, it is always helpful for users and producers of specific RM to publish these results which can help to improve the CRM itself [12].

## Documentary standards for RMs: ISO REMCO

The *ISO Committee on Reference Materials* (ISO REMCO) was established in 1975 to push for the harmonization and promotion of RMs, their production, and their application. So far, ISO REMCO developed six ISO Guides. Former ISO Guide 34 became the international standard ISO 17034 in 2016. In February 2020, it was decided to transform ISO/REMCO to an ISO technical committee. As a technical committee, ISO REMCO can review the scope and purpose of the REMCO Guides and decide to transform the guides into international standards [13]. In December 2020, the ISO Technical Management Board approved the establishment of ISO/TC 334 “*Reference materials*” with the scope “*Standardization in the competent production and use of reference materials, including the concepts, terms and definitions related to reference materials*.” These decisions were approved in November 2021. It is planned to also transform ISO Guides 30, 31, 33, and 35 into the ISO standards ISO 33400 to ISO 33405 until the end of 2023. ISO Guide 80 will become ISO 33402 until the end of 2023. Further documents on the preparation of inorganic and organic high-purity RMs are under development as ISO active work items 33406–33408.

## Recent RM trends in selected areas of application

### Additive manufacturing

Additive manufacturing processes (3D printing) are more and more important for the production of metal alloy parts. These production processes typically use a metal alloy powder that is melted into 30–50-um-thick layers in a 3D metal printer using a laser. This technology is increasingly used, e.g., in the aerospace and medical industries. As the mechanical properties of the printed parts depend on the chemical composition of the powder used, assessing the quality of the powder material is an important step in the QC of these processes. There are already some CRMs based on powders for 3D printing available from different CRM producers like steel, Al-alloy, and Ti-alloy [14, 15].

Additive manufacturing (AM) can also be applied to CRM production. Sio et al. described the preparation of a platinum group element (PGE) RM with additive manufacturing utilizing a silica matrix. They used monodispersed submicron PGE–doped silica particles produced by Stöber synthesis as feedstock materials for electrophoretic deposition (EPD). This new RM is intended for use for calibrating laser ablation inductively coupled plasma mass spectrometry (LA-ICP-MS) instruments, substituting the NIST glasses SRMs 610–617 [16]. NIST considers also producing ceramic CRMs in connection with specific ceramics AM fabrication routes. In the future, e.g., the production of such an SRM is planned after preceding round-robin studies [17]. BAM can already make metal-based test specimens by AM, which will be assessed in interlaboratory tests. RM production will then be the next step [18].

Metallic RMs used for solid-state analytical methods such as spark-emission spectrometry (SOES) and X-ray fluorescence spectrometry (XRF) are currently mostly produced by casting rods or cylinders. These materials often show undesired segregation, resulting in a variation in the chemical composition from the edge to the center of the cylinder or over the length of the rods. Segregation can be avoided if hot-isostatic pressing is used instead for the preparation of solid disk RMs. Printed samples are also expected to show a better homogeneity than casted samples, since large-scale solidification processes are avoided that favor segregation. However, possible differences in the sparking behavior of printed and cast specimens must be assessed.

### Food reference materials

Every year, tens of millions of people around the world become ill with foodborne diseases. Facing a growing population and advancing climate changes, the provision of safe food is a key challenge of the twenty-first century. The need to provide safe food has been taken up by international food safety authorities, food industry, consumer protection agencies, and policy makers such as the European Union (EU). This triggered EU to enforce regulations and directives that govern food safety. These regulations and directives need to be underpinned by suitable analytical tools for efficient implementation and CRMs for method validation and performance control. Compliance with maximum levels, but also information on nutritional value or origin, are ensured using reliable analytical methods. However, despite the general awareness, established maximum levels for critical food contaminants, and quality requirements for testing laboratories, there is a lack of urgently needed CRMs for the food sector. Current driving forces for the development of food CRMs are the compliance with legal limits, prevention of health hazards and of economic losses, the improvement of food production, and storage and transport technologies as well as the validation of methods used for food analysis.

The development and production of high-quality food CRMs require a considerable investment in money, time, and human resources. Even if a need for such a complex CRM is identified, its production is too costly for commercial RM producers. Therefore, to support industry and to ensure consumer safety, the public sector enables provision of crucial CRMs by funding their RM producers. Of special interest are contaminants that pose a global threat to food safety such as mycotoxins, pesticides, or heavy metals. In addition to (trace) elements, most of the ongoing food projects of publicly funded RM producers are dedicated to mycotoxins [19].

A similar trend can be observed in the new strategy of the *Organic Analysis Working Group* (OAWG) of the *Consultative Committee for Amount of Substance: Metrology in Chemistry and Biology* CCQM [20]. Here, the food sector has the highest priority ahead of the environmental and clinical sectors. Three key classes of compounds related to food safety are presently in the focus of OAWG: mycotoxins, pesticides, and veterinary drug residues. Relevant agricultural commodities are cereals, fruits, and vegetables, animal and plant proteins, dairy products, honey, nuts, and oils. Meanwhile, identified needs for future CRMs include chemical contaminants and residues as well as their migration from packaging, food processing, and metabolites and RMs for microbiological contaminants, authenticity, and adulteration [21, 22]. Other CRMs in demand are food CRMs for species identification based on DNA sequencing, for determining nanoparticles or microplastics in food and RMs for allergens [18]. Also, highly traded and consumed foods contaminated with toxic and/or regulated substances are a focus of future CRM developments. This includes broadly consumed beverages like coffee, tea, and cocoa. Thus, CRMs for, e.g., pesticides in tea, heavy metals in cocoa, and mycotoxins in coffee, gain in importance for analytical quality assurance [22].

In the food sector, mycotoxins are of particular concern as they are among the most prevalent and, at the same time, most dangerous contaminants in terms of chronic toxicity [23]. Estimates of the proportion of food affected by mycotoxins worldwide vary widely, ranging from about 25% for values above the legal limits to 60–80% for contamination levels above the detection limits [24]. Mycotoxins rank first in annual notifications of the Rapid Alert System for Food and Feed (RASFF) and are responsible for most rejections at European borders in recent years [25]. Due to their frequent occurrence and toxicological relevance, maximum tolerance levels for mycotoxins in food have been established [26]. Cereals, which are the world’s most important staple food with an annual harvest of about 2.8 billion tons, are considered one of the main sources of mycotoxin contamination. Along with wheat and rice, maize, the most harvested cereal, is also the one that is most susceptible to mycotoxin contamination. Therefore, CRMs for (multi)mycotoxins in maize [27] and maize products such as maize germ oils [28] will become important in the future. Also, CRMs for infant food [29] are steadily gaining in importance and CRMs for newly regulated contaminants such as ergot alkaloids.

Other frequent sources of food contamination are substances from packaging materials, industrial products, and environmental pollution. Such contaminants can enter food through direct migration or via soil, water, and air. It is therefore not surprising that contaminants such as per- and polyfluoroalkyl substances (PFAS), mineral oil saturated/aromatic hydrocarbons (MOSH/MOAH), endocrine-disrupting compounds (EDC), polycyclic aromatic hydrocarbons (PAH), brominated flame retardants (BFR), and microplastics are in the focus of activities in the environmental and food sectors [30]. Moreover, not only matrix CRMs are needed, but also calibration standards for typical methods used in food analysis that meet the ISO/IEC 17025 requirements for metrological traceability [20]. With the increasing use of LC–MS(/MS) for food analysis, also the development of isotope-labeled standards is increasingly important for an improved measurement accuracy.

### Gaseous CRMs

Gas CRMs considered here are gas mixtures in cylinders, namely measurement standards and not reference procedures or instruments such as devices to continuously dose a gas into a gas stream (e.g., permeation systems or dilutors). Compared to solid CRMs (like soil), gaseous CRMs usually have two unique properties. First, the matrix is rather simple and commonly fully characterized. Secondly, for common applications utilizing sample volume > 1 µL at ambient conditions, homogeneity does not present a challenge. However, as with other CRMs, depending on the components involved, stability can be a serious issue. Gaseous CRMs are typically produced synthetically from sufficiently pure compounds. Certification of collected real samples might be applicable in special cases.

Compared to other CRMs, the production of gaseous CRM is highly standardized, basically by the work of the ISO TC 158 group “*Gas Analysis*.” Recent developments in standardization include ISO 19229 [31] dealing with purity statements close to physical boundaries (purity cannot exceed 100%) using unsymmetrical uncertainties. Another development of ISO/CD 6142–2 [32] is the extension of the existing ISO 6142–1 [33], dealing with individual preparation and verification of each gas cylinder to batch certification and application of generic uncertainties. As with other CRMs, the demand for gaseous CRMs is much larger than the number of actually available CRMs.

Current trends of gaseous CRMs include the reduction of measurement uncertainties and the improvement of detection sensitivity. A reduction of the uncertainty is a general requirement for CRM production. For gaseous CRMs in environmental analysis, a prominent driver is the data quality directive of the *Global Atmospheric Watch Program of the World Meteorological Organisation* (WMO GAW), which requires an expanded target uncertainty of, e.g., 0.1 nmol/mol for N_2_O [34]. In general, lower uncertainties make a CRM more valuable. Main drivers for CRMs with lower certified amount fractions are environmental measurements (e.g., volatile organic compounds (VOC) in clean air) and purity analysis for very pure gasses (e.g., H_2_ purity according to ISO 14587 [35]) or even zero gasses (uncontaminated air) in environmental analysis. At very low concentrations, the stability of CRMs becomes a serious issue. In some cases, CRMs can only be prepared as higher concentrated premixtures for further online dilution.

Other trends include the development of CRMs for reactive gasses such as HCl, NO_*x*_, NH_3_, sulfur compounds, formaldehyde, and many other compounds that are increasingly relevant at immission or emission levels. This goes together with the development of surface treatment and coatings of gas cylinders. A generic field of interest is also moisture not only at low concentrations but in a variety of matrices. Concerning gas CRMs for purity, an ongoing activity triggered by the climate change and the interest in greener energy resources is the development of hydrogen CRMs to meet the challenging demands defined by ISO 14687 for reactive gasses at low contents (e.g., 4 nmol/mol for total sulfur). There is evidence that sufficiently stable CRMs might not be achievable for these specification levels. This can be overcome by CRMs in the form of more concentrated mixtures that have to be further diluted online. Due to hydrogen storage in the existing natural gas grid, CRMs for mixtures of natural gas with up to 30% of hydrogen are developed.

Another driver for new gaseous CRMs is the decarbonization of the energy gasses. For carbon capture storage and use, CRMs for impurities in gaseous and liquefied carbon dioxide are increasingly being developed. Depending on the area of application, with focus on permanent gases, stripper compounds (e.g., amines) or other relevant impurities such as toxic substances relevant for use in food and beverage applications. There is also still a demand for CRMs for biomethane that can contain a variety of siloxanes, sulfur compounds, and VOCs originating from the biological production process.

Moreover, CRMs for trading of liquefied natural gas (LNG) and liquefied petroleum gas (LPG) are required by the market.

Finally, CRMs are further needed for reliable material data generation as required, e.g., for the development of equations of state models as needed for gas transport and processing.

In general, for many technological applications, CRMs for gas mixtures and “pure compounds” that are fully characterized regarding the impurities and concerning identity and content are needed. These demands range from uncommon components such as halogen-free refrigerator or isolation gases via noble gases to other gas mixtures, even explosive ones. As gas analysis by advanced molecular spectrometry methods like cavity ring down spectrometry (CRDS), which provide a high sensitivity, can depend on the isotopic composition, there is also an increasing demand for gaseous CRMs with stated chemical and isotopic composition. Quantification of small differences in isotopic composition can also be used to trace back the origin of samples and for authentication purposes.

### Reference materials for optical spectroscopic methods

Currently, there are increasing activities on the standardization of fluorescence-based methods such as fluorescence spectroscopy and fluorescence microscopy. These are among the most frequently used optical spectroscopic methods in the life and material sciences because of the high sensitivity and multiparametric nature of fluorescence. This is largely related to the considerable influence of instrument-specific effects on fluorescence signals. This hampers the comparability of fluorescence data across different instruments and laboratories and renders quantification from measured fluorescence intensities challenging [36, 37]. Current trends in fluorometry include the renaissance of fluorescence quantum yield measurements of transparent and scattering fluorescent samples [38]. The fluorescence quantum yield presents one of the key properties of molecular and nanocrystalline emitters and fluorescent particles [38]. This triggered the development of the standard IEC 62607–3-1 *Nanomanufacturing – Key control characteristics—Part 3–1: Luminescent nanomaterials – Quantum efficiency* released by the International Electrotechnical Commission (IEC) in 2014. This has created a need for certified fluorescence quantum yield standards. Therefore BAM released the first set of twelve certified quantum yield standards BAM-F011 and BAM-F013–BAM-F023 in 2021. These CRMs, which cover the ultraviolet (UV), visible (VIS), and near-infrared (NIR) spectral regions, can be used for the reliable relative determination of fluorescence quantum yields of transparent fluorescent samples and the evaluation of the performance of integrating sphere setups employed for absolute quantum yield measurements.

Another trend is the increasing interest in fluorescence measurements in the short-wave infrared (SWIR) above 1000 nm, particularly in the bioimaging and medical area. This enables optical measurements with a higher penetration depth and a higher sensitivity or better signal-to-background ratio due to a strongly reduced autofluorescence from tissue components. Also, due to the strongly reduced scattering, a much better spatial resolution can be achieved [39]. In the future, reliable measurements in this wavelength region will require suitable spectral fluorescence standards for instrument calibration or its control as well as fluorescence intensity or quantum yield standards for the determination of this key performance parameter of SWIR emissive luminophores and optical contrast agents [40]. In addition standardized methods as well as suitable RMs and CRMs will be needed for industrially relevant fluorescence measurements like the characterization of emitters used for the production of optical devices such as light-emitting diodes (LEDs) and LED converter materials, and for applications in solid-state lighting like plasma displays.

In 2020, in the area of broadly used light microscopy, the community-driven initiative *Quality Assessment and Reproducibility for Instruments & Images in Light Microscopy* (QUAREP-LiMi) was started to improve the reproducibility for light microscopy image data through QC management of instruments and images [36]. This international network is driven by imaging facilities and supported by instrument manufacturers, national metrology institutes, and standardization organizations. QUAREP-LiMi aims for a common set of quality assurance and control guidelines for hardware calibration and image acquisition, management, and analysis. This initiative is currently developing a set of documents and measurement protocols on these topics, together with recommendations on suitable calibration tools and fluorescence standards for wide field and confocal microscopy as well as super-resolution microscopy [41–43]. This will also trigger the development of new RMs and CRMs.

### Nano reference materials

In the last three decades, the interest in nanoscale reference materials considerably increased triggered by the vastly developed nanotechnology and the important role of nanomaterials for key technologies of the twenty-first century. This set also the focus of standardization organizations like ISO and IEC on standardizing an increasing number of analytical and physico-chemical methods, which are particularly relevant for the characterization of nanomaterials. This led, e.g., to the establishment of ISO TC 229 *Nanomaterials* and, in addition, to the *Working Party on Manufactured Nanomaterials* (WPMN) as part of the *Organization for Economic Cooperation and Development* (OECD). While standardization is relatively rapidly developing [44], only a small number of nanoscale CRMs (nanoCRMs) are meanwhile available. This includes layered structures and ideal, i.e., spherical particles of different sizes commonly made from gold or polystyrene [45].

The high complexity of the production of nanoscale RMs and their often limited stability of only several months, in conjunction with frequent difficulties to reproduce and recertify these nanomaterials, are imposing considerable challenges on producers of nanoRMs. This situation encouraged nanoRM producers to rely on highly stable spherical nanomaterials like gold, polystyrene, and silica or identify other possibilities for generating nanoRMs.

A commonly used alternative for nanoRMs are proficiency test materials [2]. In this case, evidence for stability is gained during the lifetime of the material from the different characterization results. While this option is commonly used in the scientific community and meets most of its requirements, it does not fulfill the requirements on materials suitable for calibration or method validation purposes in the regulatory context. Another commonly used option is the production of nanoRMs on demand. Thereby, batches of chemicals to produce nanomaterials are stored under controlled conditions and the nanomaterial production is done on demand following a defined, detailed, and reliable procedure. This approach gained attention during the last years.

The actual technical and regulatory developments influence the demands for nanomaterials. In the beginning of 2020, the REACH regulations were extended with annexes for nanomaterials [46]. Regulation is asking for robust and reliable results for the registration of nanomaterials. In the case of the equivalent circular diameter, this implies a characterization with at least two independent methods, based on different measurement principles. In most cases, this will include an imaging method like electron microscopy and a method with a high counting rate like centrifugal liquid sedimentation or a differential mobility analyzing system. Besides a reliable equivalent circular diameter, properties like surface area, morphology, solubility, surface reactivity, and structure (e.g., core–shell) are also requested. Therefore, measurement protocols for methods, which can provide these features, are in the focus of the work of ISO TC 229 [47] and OECD-WPMN [48, 49]. This is an important development for the provision of reference materials, reference methods, and reference data. It is not sufficient to provide ideal spherical nanoparticles. Reference nanoparticles with a specific shape and surface morphology are required to reliably calibrate instruments. For applications in environment and human toxicology, the surface reactivity and the solubility of materials are important parameters. These parameters need to be monitored by positive or negative controls, requiring suitable nanoRMs.

In the next years, the demands on nanoRMs will probably shift to include more parameters. Nanomaterials are additives in several other materials (e.g., additive manufacturing) and the basis for more complex materials like multiscale structured materials. These developments will probably lead to further regulation (e.g., transport, end of life, food contact materials) and will increase the need for more complex nanoRMs and nanoCRMs.

### Geochemical reference materials

The economic development and management of environmental and geological risks such as anthropogenic pollution, climate change, or volcanic hazards rely on the knowledge of the chemical composition of geomaterials. Sound political and economic decision-making depends to a large extent on the availability of well-characterized geological RMs (geoRMs), as basis for the generation of robust, at best SI-traceable, analytical data. GeoRMs mainly include rocks and minerals of different chemical compositions. Most of these materials are predominantly inorganic, but to a very small extent, also organic materials such as coal or oil shale occur. GeoRMs have been and continue to be produced mainly by a few government institutions, commercial companies, and scientific associations. For geological RMs, it is common practice that secondary characterization studies increase the catalog of elemental concentrations and isotope ratios which were not certified or characterized during the initial characterization of the RM. Such secondary characterization data are collected in the *GeoRem* database [10]. In addition to individual data, the *GeoRem* database provides compiled elemental concentrations and isotopic ratios when sufficient robust data are available.

Rock RMs exist for all major rock classes, including magmatic, metamorphic, and sedimentary rocks. Particularly, ore RMs are of great economic importance and enjoy strong demand from the mining industry. At least a small number of different rock RMs already exist for each of the major rock classes. However, to cover a wider range of rock types, new rock RMs are needed in the future. Rock RMs are typically offered as powders ground to a small grain size (e.g., < 63 µm) to overcome the naturally occurring heterogeneity of the multimineral rock matrix. The chemical composition of rock RMs is often certified or characterized only for major elements and some highly concentrated trace elements. Isotopic ratios or isotopic composition have never been certified for a rock RM, except for some carbonates used for stable isotope RMs.

Since the development of analytical techniques for the in situ analysis of elements and isotopes in geological samples such as LA-ICP-MS, µm X-ray fluorescence spectroscopy (µm-XRF), laser-induced breakdown spectroscopy (LIBS), and secondary ion mass spectrometry (SIMS), there has been a growing demand for homogeneous RMs made of glass, minerals, or pressed pellets. The NIST SRM 600 glasses are the most popular glass reference materials [50]. However, these glasses have a non-natural composition and are therefore not suited for a matrix-matched calibration. To overcome this challenge, Max-Planck-Institute’s DING glasses were made from natural rocks. These glasses are well characterized in terms of major and trace element concentrations and some isotopic ratios [51] but are only available on request and in very small quantities. In the future, there is an urgent need of the growing community of in situ analysts for homogeneous glass reference materials of natural geological composition.

A relatively new approach to provide calibration materials for in situ analysis is the production of pressed rock and mineral reference materials in the form of nanopellets [52]. To overcome the natural heterogeneity of rocks, the materials are ground to a grain size of less than 1 µm. This grain size reduction is usually suitable for most in situ techniques. However, depending on the spot sizes used (amount of material sampled/sample size) or vacuum or surface requirements, nanopellets should be employed with caution.

Homogeneous monomineralic RMs are also in demand for in situ analysis. Due to the formation processes of minerals in the magmatic, metamorphic, or sedimentary environment, natural minerals usually do not provide a homogeneous distribution of elemental concentrations and isotopic ratios. Therefore, the preparation of monomineralic reference materials begins with the selection of a suitable homogeneous source material, followed by robust homogeneity testing and characterization of the target analytes. Some monomineralic RMs are already available for some important geological applications such as radiometric dating, and diffusion and zonation studies [53]. One of the best-known examples of a homogeneous monomineralic RM is the 91,500 zircon material. This zircon reference material has been characterized for its U-Th-Pb and oxygen isotope inventory [54] and is widely used for in situ zircon analyses.

## Outlook

In our article, only selected areas of the still strongly growing field of RM were summarized and the most important trends were addressed.

In the future, the number of RM manufacturers—with and without accreditation according to ISO 17034 will increase. In particular, manufacturers will emerge with a very focused portfolio of RM in contrast to many publicly funded RM manufacturers. Also, some manufacturers will concentrate on special matrices, while others will pursue a method-specific approach, e.g., RMs for microanalytics with particularly high requirements on material homogeneity. The transfer of ISO-REMCO to an ISO technical committee will broaden the normative basis of RM production. Also, the importance of RMs in terms of accreditation of manufacturers and users will increase.

As the development times for metrologically high-quality CRMs are often too long, in the future, parts of the RM characterization will be shifted to the user to shorten the time of RM availability for users. This implies that RM application by users will be utilized to gradually increase the wealth of information on the properties of the RM.

In addition to RMs that are classically produced as batch RMs, another strategy presents the production of RMs on demand. This can be done by emerging technologies that provide a very high degree of reproducibility such as 3D printing, automated RM preparation, or even the production of RMs by the user directly before use from reagents and a validated standard operation procedure provided by the RM manufacturer.

In the area of classic RMs, the requirements will increase toward lower contents, smaller uncertainties, and additional components such as sum parameters, driven by legislative requirements. Our quality of life, including the supply of safe food, depends strongly on a healthy planet. Therefore, future CRM developments for environmental analysis will gain importance to support national and international efforts for clean water, soil, and air [and biota]. The focus will be on emerging organic pollutants such as per- and polyfluoroalkyl substances (PFAS), flame retardants, or endocrine-disrupting compounds, as well as inorganic pollutants such as heavy metals. Another trend presents multifunctional RMs in emerging technology fields such as energy research, biotechnology, nanotechnology, and nanobiotechnology.
